# Validation of the German HeartQoL: a short health-related quality of life questionnaire for cardiac patients

**DOI:** 10.1007/s11136-019-02384-6

**Published:** 2019-12-12

**Authors:** Alexandra Huber, Neil Oldridge, Werner Benzer, Hugo Saner, Stefan Höfer

**Affiliations:** 1 Department of Medical Psychology, Medical University of Innsbruck, Innsbruck, Austria; 2 College of Health Sciences, University of Wisconsin-Milwaukee, Milwaukee, WI USA; 3 Outpatient Cardiac Rehabilitation Centre, Feldkirch, Austria; 4 Preventive Cardiology and Sports Medicine, Swiss Cardiovascular Centre Bern University Hospital, Inselspital, Bern, Switzerland

**Keywords:** Health-related quality of life, German HeartQoL questionnaire validation, Ischemic heart disease, Angina, Myocardial infarction, Heart failure

## Abstract

**Purpose:**

The aim of this study was to evaluate psychometric properties of the core disease-specific 14-item German HeartQoL questionnaire.

**Methods:**

As an extension of the international HeartQol Project, cross-sectional and longitudinal health-related quality of life (HRQL) data were collected from 305 patients with angina (*N *= 101), myocardial infarction (*N *= 123), or ischemic heart failure (*N *= 81) in Austria and Switzerland using German versions of the HeartQoL, the Short Form-36 Health Survey (SF-36), and the Hospital Anxiety and Depression Scale. The underlying factor structure was examined with Mokken Scaling analysis; then convergent, divergent, and discriminative validity, internal consistency reliability, and responsiveness were assessed.

**Results:**

The highest HRQL scores were reported by patients with myocardial infarction followed by ischemic heart failure and then angina. The two-factor structure was confirmed with strong physical, emotional, and global scale *H* coefficients (> .50). Divergent and convergent validity (from *r *= .04 to .78) were shown for each diagnosis; discriminative validity was verified as well (partially: age, sex, and disease severity; largely: SF-36 health status/transition; totally: anxiety and depression). Internal consistency reliability was excellent (Cronbach’s alpha = .91). In terms of responsiveness, physical and global scale scores improved significantly after percutaneous coronary intervention (*p *< .01) while after cardiac rehabilitation all scale scores improved significantly (*p *< .001).

**Conclusions:**

The German HeartQoL questionnaire is a valid and reliable HRQL instrument with these data supporting its potential use in clinical practice and research to assess and compare HRQL in German-speaking patients with ischemic heart disease. The shortness of the tool may prove to be helpful particularly in clinical practice.

## Background

Health-related quality of life (HRQL) is acknowledged as an important outcome measure of health care, particularly in patients with chronic diseases such as cardiovascular diseases [1–3]. According to the World Health Organization, cardiovascular diseases were the leading cause of death globally in 2016, accounting for 31.4% of all deaths with 52.8% of cardiovascular diseases due to ischemic heart disease (IHD) [4]. Latest available domestic data showed slightly decreasing numbers of deaths caused by IHD for German-speaking countries including Austria (16.8% in 2017) [5], Germany (13.9% in 2015) [6], and Switzerland (10.9% in 2015) [7]. Furthermore, IHD was the second leading cause of disability-adjusted life years in Western Europe in 2010 [8], with major reductions in HRQL and estimated health care costs (in-patient stays) of, for example, 359 million Euros per year in Austria [9]. Patient-reported outcomes, including HRQL, are predictive of mortality, cardiovascular events, rehospitalization, and care expenses in patients with cardiovascular diseases [10, 11]. As a result, patient-reported outcomes have been recommended for routine use in clinical practice [1, 2].

Generic and specific HRQL questionnaires have been developed and validated to measure short- and medium-term changes in terms of patient-reported HRQL. Generic instruments allow comparisons of HRQL between different healthy populations, between different diseases and healthy populations, and populations with various diseases. The Short Form-36 Health Survey (SF-36) [12, 13] is one of the most widely used generic HRQL measures in the general population and patients with cardiovascular disease [14–16]. In contrast, disease-specific HRQL questionnaires are designed to provide more tailored information concerning a given disease or a specific diagnosis [17] and therefore offer greater clinical relevance than generic questionnaires. But this approach may preclude between-diagnosis HRQL outcome comparisons within a given disease. Core disease-specific HRQL instruments, like the HeartQoL questionnaire [18, 19], provide a potential solution to this limitation. The HeartQoL was developed and validated in the HeartQoL Project with 6384 patients with IHD between 2002 and 2011 who lived in five global regions with a total of 22 countries where 15 languages are spoken [18, 19]. With permission, and in a cross-sectional survey [18], 14 items from the Seattle Angina Questionnaire (SAQ) [20], the MacNew Heart Disease Health-related Quality of Life Questionnaire (MacNew) [21], and the Minnesota Living with Heart Failure Questionnaire (MLHFQ) [22] were identified and comprise the HeartQoL. In the second step, the psychometric properties of the 14-item HeartQoL were tested [19].

When HRQL questionnaires are used as outcome measures, they need to demonstrate reliability (the degree to which an instrument is free from random error), validity (the degree to which the instrument really measures what it purports to measure), and responsiveness (the ability to detect change over time) [23]. The psychometric properties of the SF-36 [24] and the three questionnaires on which the HeartQoL is based upon (SAQ, MacNew, MLHFQ) have all been individually assessed in the German language [25, 26]. Reliability, validity, and partially responsiveness of the English HeartQoL have been already demonstrated in patients with angina, myocardial infarction (MI), and ischemic heart failure in the HeartQoL Project [19], in patients with angina or MI living in the USA [27], and with reliability and validity documented in stable coronary patients in EuroAspire IV [28]. Other language versions, e.g., examining Danish-speaking patients with atrial fibrillation [29], an implantable cardioverter defibrillator [30], and valve surgery [31] or Chinese patients with angina, MI, and ischemic heart failure [submitted] demonstrated adequate psychometric properties as well.

## Methods

The aim of this study was to determine the psychometric properties of the German HeartQoL. Cross-sectional (*N *= 305) and longitudinal data (*N *= 184) were collected from five centers in Austria and Switzerland to validate the German version of the HeartQoL in patients with angina, MI, and ischemic heart failure. The German HeartQoL, SF-36 [24] and Hospital Anxiety and Depression Scale (HADS) [32] were administered as paper and pencil questionnaires.

### Patients

Patients diagnosed with angina, MI, or ischemic heart failure were recruited at five sites with ethics approval. Eligibility criteria were the same as in the original HeartQoL project and included the following: (a) currently being treated for angina (disease severity: Canadian Cardiovascular Society (CCS) class II, III or IV) [33] with an objective measure of IHD; (b) had experienced a documented MI between one to six months previously; (c) currently being treated for ischemic heart failure (disease severity: New York Heart Association (NYHA) class II, III, or IV) [34] with evidence of a left ventricular dysfunction and an objective measure of IHD. Additional eligibility criteria included the following: age ≥ 18 years, to be able to complete the self-administered battery of HRQL measurement in German, no hospitalization in the last 6 weeks, and no serious psychiatric disorder as well as no current substance abuse as identified by the referring physician. Participation in the study was discussed with all patients meeting the eligibility criteria and written informed consent was obtained from those agreeing to participate.

### Patient-reported questionnaires

#### Sociodemographic and clinical variables

Age, sex, body mass index, comorbidities (cancer, chronic pain, dialysis, gastro-intestinal diseases, orthopedic diseases, neurological diseases, respiratory diseases, and urogenital diseases), diabetes, hypercholesterolemia, hypertension, physical inactivity, and smoking, shortness of breath, and chest pain were self-reported by patients with disease severity evaluated by physicians for patients with angina (CCS class II, III, or IV) and ischemic heart failure (NYHA II, III, or IV) diagnoses.

#### HeartQoL

The German version of the disease-specific HeartQoL was used to measure HRQL in this study. The questionnaire consists of a physical (10 items) and an emotional (4 items) subscale making up the 14-item global scale with higher values representing better HRQL [18]. The HeartQoL items are based on the items in the SAQ, MacNew, and MLHFQ (25, 26) which were translated into the German language. All items on the physical (e.g., “In the last 4 weeks, have you been bothered by having to lift or move heavy objects?”) and the emotional subscale (e.g., “In the last 4 weeks, have you been bothered by being worried?”) are answered on a 4-point scale ranging from “bothered a lot” (= 0) to “not bothered” (= 3).

#### Hospital Anxiety and Depression Scale (HADS)

The German version of the HADS [32] is a 14-item self-assessment questionnaire used to screen for anxiety and depressive symptoms with an anxiety (e.g., “I get a sort of frightened feeling as if something awful is about to happen”) and a depression subscale (e.g., “I look forward with enjoyment to things”). The items are answered on a scale ranging from 0 to 3 with higher scores representing higher levels of anxiety or depression. In particular, patients with a cut-off score ≥ 8 are considered to be potentially either anxious or depressed in terms of a clinical diagnosis [35]. Moreover, as some studies argue for a general distress factor summing up all anxiety and depression symptoms, a respective common score including all items was generated with cut-off criteria of ≥ 12 showing the best sensitivity/specificity for general distress in patients with coronary heart disease [36]. Cronbach’s alpha in the total cohort was .81 for both scales and .87 for the whole instrument.

#### SF-36 Health Survey (SF-36)

The German version of the SF-36 [24] consists of 36 items, each scored in one of eight scales which form two distinct summary measures, namely the Physical Component Summary (PCS) and the Mental Component Summary (MCS) measure. Data from the PCS and MCS are presented as *T*-scores with a mean (M) of 50 ± 10 standard deviation (SD), with higher scores indicating better HRQL. The first item “In general, would you say your health is…excellent/very good/good/fair/poor?” and the second item “Compared to 1 year ago, how would you rate your health in general now? Much better/somewhat better/about the same/somewhat worse/much worse… than 1 year ago” were used to check the discriminative validity of the HeartQoL. Cronbach’s alpha in the total cohort was .88 for PCS and .91 for MCS.

#### Statistical analyses

Only data sets with full information on the cardiac diagnoses were included in the cross-sectional (*N *= 305) and longitudinal statistics (*N *= 184). No outliers were detected (*z*-transformed means exceeding ± 3.29) and no data imputations were carried out. Mean ± SD and proportions for the total cohort and each of the three IHD diagnoses (angina, MI, and ischemic heart failure) were identified. Categorical sociodemographic and clinical variables were analyzed with Pearson’s *Chi*-square test while continuous variables and scale means were examined with analyses of co-variance (ANCOVA with post hoc Bonferroni correction as they were adjusted for age, sex, risk factors, and disease severity within diagnosis). A two-sided *p*-value < .05 was considered as significant. Data were analyzed using IBM SPSS Statistics 24 [37] and STATA 14 [38].

The evaluation of the psychometric properties of the German HeartQoL followed criteria recommended by the Scientific Advisory Committee [23]. Floor and ceiling effects of the HeartQoL were considered present when more than 15% patients of the total group and of each diagnosis reported the lowest score (= 0; floor) or the highest (= 3; ceiling) score [39]. Mokken scale analysis was used to determine the scale structure. Loevinger’s *H*_*i*_ coefficients for each item as well as *H* coefficients for the global scale and each subscale were calculated with a cut-off value of ≥ .50 considered a “strong,” .49–.40 a “moderate,” and .39–.30 a “weak” Mokken scale [40]. Internal consistency reliability was measured using Cronbach’s alpha with values of ≥ .70 acceptable for group and ≥ .90 for individual comparisons [41]. Convergent and divergent validity were tested with Pearson’s coefficient inter-correlations which can be interpreted as follows: *r* < .10 = no correlation, *r* = .10–.29 = low correlation, *r* = .30–.49 = moderate correlation, *r* ≥ .50 = high correlation [41]. These correlations were then compared using Steiger’s test [42]. The “known-groups” approach [43] was used to test for discriminative validity on groups known based on previous research and clinical knowledge to differ on the variables of interest. Discriminative validity was tested with the following groups: significantly higher HeartQoL scores were hypothesized to be reported by patients with either MI or angina than by patients with ischemic heart failure and, regardless of diagnosis, by younger and by male patients than by older and female patients, by patients reporting “excellent/very good” compared to “good” or” fair/poor” SF-36 general health status, by patients reporting “improved” compared to either “no change” or “deteriorated” health on the SF-36 health transition item, by patients not exceeding the cut-off score of ≥ 8 (anxiety or depression) or ≥ 12 (general distress) compared to those exceeding these scores, and by patients with angina or ischemic heart failure with less disease severity than those with greater severity.

Responsiveness was tested in two different groups. Patients undergoing percutaneous coronary intervention (PCI) were assessed pre and four weeks post the intervention and patients participating in a four-week in-patient cardiac rehabilitation (CR) program completed the questionnaires at the start and end of the program. Results are reported as effect sizes (small: ≥ .20 to < .50; moderate: ≥ .50 to < .80; and large: ≥ .80) using the standardized response mean method (effect size = mean score time 2 − mean score time 1/SD of the change score) [44].

## Results

### Patient characteristics and questionnaire means

In this analysis, 101 patients (33.1%) had documented angina, 123 (40.3%) had a documented MI, and 81 (26.6%) had documented ischemic heart failure. None of the sociodemographic or patient data were significantly different in patients from the two countries. The mean age of the total cohort was 63.5 ± 11.1 years and 77.7% were male, being representative for a German-speaking cardiac population with IHD [45–47]. Most of the patients were married (71.1%) and did not complete high school (62.6%), and about a half of them were employees (white collar; 52.1%). Physical inactivity was the most prevalent risk factor (60.7%), followed by hypercholesterolemia (55.4%), and hypertension (50.8%). Among patients with angina, 56.1% were classified as CCS class II and 46.9% of patients with ischemic heart failure were classified as NYHA class II; all other patients were classified in either class III or IV. Only 41 patients (13.4%) suffered from comorbidities, e.g., cancer, chronic pain, and so on. Anxiety scores ≥ 8 were reported by 30.2% and depression scores ≥ 8 by 20.7% of the total cohort with the greatest number of either anxious or depressed patients observed in patients with angina. General distress scores ≥ 12 were reported by 38.0% of all cardiac patients with almost half of the patients with angina feeling distressed. All sociodemographic and clinical characteristics are detailed in Table 1 as well as mean ± SD for each questionnaire.

**Table 1:** Description of the sample

	Total cohort (*N *= 305)	AP (*N *= 101; 33.1%)	MI (*N *= 123; 40.3%)	HF (*N *= 81; 26.6%)	*p*-value
**Demographics**					
Age	63.46 ± 11.1	66.06 ± 9.91	62.19 ± 11.89	62.20 ± 10.85	< .05^a,c^
Female	65 (21.3%)	24 (23.8%)	31 (25.2%)	10 (12.3%)	< .05^b^
Male	237 (77.7%)	76 (75.2%)	92 (74.8%)	69 (85.2%)	n.s.
**Marital status**					
Single	51 (16.7%)	13 (12.9%)	23 (18.7%)	15 (18.5%)	n.s.
Married/partnership	217 (71.1%)	81 (80.2%)	83 (67.5%)	53 (65.4%)	< .05^a,c^
Other	32 (10.5%)	5 (5.0%)	16 (13.0%)	11 (13.6%)	< .05^a,c^
**Education**					
< High school	191 (62.6%)	64 (63.4%)	76 (61.8%)	51 (63.0%)	n.s.
= High school	40 (13.1%)	11 (10.9%)	15 (12.2%)	14 (17.3%)	n.s.
> High school	44 (14.4%)	13 (12.9%)	23 (18.7%)	8 (9.9%)	< .05^a^
**Employment**					
Blue collar	73 (23.9%)	25 (24.8%)	26 (21.1%)	22 (27.2%)	n.s.
White collar	159 (52.1%)	46 (45.5%)	69 (56.1%)	44 (54.3%)	n.s.
**Risk factors**					
BMI	26.98 ± 4.71	26.61 ± 4.59	27.02 ± 4.72	27.38 ± 4.88	n.s.
Comorbidities^†^	41 (13.4%)	21 (20.8%)	4 (3.3%)	16 (19.8%)	< .001^a,b^
Diabetes^§^	44 (14.4%)	11 (10.9%)	15 (12.2%)	18 (22.2%)	< .05^c^
Hypercholesterolemia^§^	169 (55.4%)	65 (64.4%)	62 (50.4%)	42 (51.9%)	< .05^a^
Hypertension^§^	155 (50.8%)	55 (56.4%)	64 (52.0%)	36 (44.4%)	n.s.
Physical inactivity^#^	185 (60.7%)	57 (56.4%)	84 (68.3%)	44 (54.3%)	< .05^b^
Smoking	43 (14.1%)	11 (10.9%)	19 (15.4%)	13 (16.0%)	n.s.
**Interventions**					
PCI	113 (37.0%)	60 (59.4%)	35 (28.5%)	18 (22.2%)	< .001^a,c^
CR	166 (54.4%)	41 (40.6%)	88 (71.5%)	37 (45.7%)	< .001^a,b^
**Disease severity**					
CCS ≤ II	57 (56.4%)	57 (56.4%)	–	–	–
CCS III and IV	42 (41.6%)	42 (41.6%)	–	–	–
NYHA ≤ II	38 (46.9%)	–	–	38 (46.9%)	–
NYHA III & IV	40 (49.4%)	–	–	40 (49.4%)	–
**Questionnaire scores**					
*Heart QoL*					
Physical subscale	1.81 ± .72	1.65 ± .73	1.97 ± .70	1.79 ± .70	< .05^a^
Emotional subscale	2.20 ± .74	2.21 ± .72	2.29 ± .74	2.18 ± .74	n.s.
Global scale	1.92 ± .64	1.80 ± .65	2.07 ± .63	1.90 ± .63	< .05^a^
*HADS*					
Anxiety scores	5.68 ± 3.75	5.90 ± 3.51	5.47 ± 3.82	5.49 ± 3.78	n.s.
Anxious*	92 (30.2%)	35 (34.7%)	33 (26.8%)	24 (29.6%)	n.s.
Depression scores	4.85 ± 3.57	4.89 ± 3.16	4.45 ± 3.70	5.01 ± 3.86	n.s.
Depressed*	63 (20.7%)	26 (25.7%)	22 (17.9%)	15 (18.5%)	n.s.
General distress scores	10.53 ± 6.62	11.15 ± 6.00	10.02 ± 6.88	10.54 ± 6.96	n.s.
Generally stressed*	116 (38.0%)	47 (46.5%)	42 (34.1%)	27 (33.3%)	n.s.
*SF-36*					
PCS	41.10 ± 9.94	39.53 ± 9.56	42.12 ± 10.54	41.56 ± 9.86	n.s.
MCS	47.02 ± 11.19	47.42 ± 10.67	46.85 ± 11.88	47.98 ± 10.76	n.s.

*p*-value between diagnosis with ANCOVAs (scores adjusted for age, sex, risk factors) post hoc Bonferroni correction (continuous variables) and *Chi*-square tests (categorical variables)

*AP* angina, *MI* myocardial infarction, *HF* ischemic heart failure, *N* number of patients, *scale* mean ± standard deviation, *BMI* body mass index, *PCI* percutaneous coronary intervention, *CR* cardiac rehabilitation; *CCS* Canadian Cardiovascular Society, *NYHA* New York Heart Association, *HADS* Hospital Anxiety and Depression Scale, *SF-36* Short Form-36 Health Survey, *PCS* physical component summary, *MCS* mental component summary

^†^Including cancer, chronic pain, dialysis, gastro-intestinal diseases, orthopedic diseases, neurological diseases, respiratory diseases, and urogenital diseases

^§^As told by his/her physician

^#^Active on < 3 occasions per week

*HADS cut-off score ≥ 8 for the anxiety/depression subscales and ≥ 12 for the general distress factor (*N* and %)

^a^AP vs. MI

^b^MI vs. HF

^c^AP vs. HF; *n.s.* not significant; data missing if sample sizes do not equal *N* or 100% for each group

For the total cohort, the mean HeartQoL physical score was 1.81 ± .72, the mean emotional score was 2.20 ± .74, and the global HRQL score was 1.92 ± .64 with ANCOVA results demonstrating significant physical and global score differences between patients with angina and MI. The highest physical and emotional HeartQoL scores were reported by patients with MI as hypothesized; however, the lowest scores were found in patients with angina (physical subscale and global scale *p *< .05) and not in ischemic heart failure (Tables 1, 2) as hypothesized. Means on the HADS or the SF-36 scales were not significant when comparing across different diagnoses (Table 1). HeartQoL floor effects on each scale were always ≤ 3.3% in the total group and in each diagnosis (Table 2). HeartQoL physical and global score ceiling effects were ≤ 3.0% in the total group and ≤ 4.9% in each diagnosis while ceiling effects on the emotional subscale were observed in 23.3% of the total group, ranging from 20.4 to 25.2% by diagnosis (Table 2).

**Table 2:** Measurement values of the German HeartQoL

	Total cohort (*N *= 302)	AP (*N *= 100)	MI (*N *= 123)	HF (*N *= 79)
**Physical subscale**	1.81 ± .72	1.66 ± .73	1.93 ± .71	1.80 ± .69
Floor effect	.7%	2.0%	0%	0%
Ceiling effect	3.0%	1.0%	4.9%	2.5%
Cronbach’s alpha	.91	.91	.92	.90
**Emotional subscale**	2.20 ± .74	2.16 ± .73	2.24 ± .75	2.19 ± .73
Floor effect	1.7%	1.0%	3.3%	0%
Ceiling effect	23.3%	20.4%	25.2%	24.1%
Cronbach’s alpha	.89	.89	.90	.89
**Global scale**	1.92 ± .64	1.80 ± .64	2.02 ± .65	1.91 ± .62
Floor effect	.7%	2.0%	0%	0%
Ceiling effect	1.7%	0%	3.3%	1.3%
Cronbach’s alpha	.92	.91	.93	.91

*AP* angina, *MI* myocardial infarction, *HF* ischemic heart failure, *N* number of patients, *scale* mean ± standard deviation, *floor effect* poorest health-related quality of life, *ceiling effect* highest health-related quality of life, *Cronbach’s alpha* internal consistency

### Psychometric properties of the HeartQoL

#### Factor structure

Mokken analysis revealed that the HeartQoL *H* coefficients were “strong” with .59 for the physical subscale, .77 for the emotional subscale, and .51 for the global scale confirming the original HeartQoL two-factor structure. The *H*_*i*_ coefficients were mostly “strong” ranging from .52 to .68 on the physical subscale and from .74 to .81 on the emotional subscale (Table 3) with the only exception being on the eighth item (“…feeling tired, fatigued, low on energy?”) where the *H*_*i*_ coefficient was .48 on the physical subscale (Table 3).

**Table 3:** Mokken Scale analysis of the German HeartQoL

	Loevinger’s *H* coefficients		
	Physical *H*_*i*_ (*N* = 260)	Emotional *H*_*i*_ (*N* = 287)	Global *H*_*i*_ (*N* = 255)
1. Walk indoors on level ground?	.52		.44
2. Garden, vacuum, or carry groceries?	.63		.53
3. Climb a hill or a flight of stairs without stopping?	.65		.56
4. Walk more than 100 yards at a brisk pace?	.59		.51
5. Lift or move heavy objects?	.60		.51
6. Feeling short of breath?	.54		.49
7. Being physically restricted?	.61		.56
8. Feeling tired, fatigued, low on energy?	.48		.53
9. Not feeling relaxed and free of tension?		.74	.46
10. Feeling depressed?		.76	.43
11. Being frustrated?		.78	.46
12. Being worried?		.81	.47
13. Being limited in doing sports or exercise?	.59		.53
14. Working around the house or yard?	.68		.61
HeartQoL *H*	.59	.77	.51

#### Reliability

Internal consistency reliability was confirmed with Cronbach’s alpha ranging from .89 to .92 in the total cohort, from .89 to .91 in patients with angina, from .90 to .93 in patients with MI, and from .89 to .91 in patients with ischemic heart failure (Table 2).

#### Validity

- Convergent validity was confirmed in the total cohort and each diagnosis with correlations between the HeartQoL and SF-36 physical scales ranging from .62 to .78 and from .71 to .76 between the HeartQoL and SF-36 emotional scales. Correlations between dissimilar scales of both instruments were significantly lower according to Steiger’s test for comparing Pearson correlations (Table 4).

**Table 4:** Convergent validity of the German HeartQoL with the SF-36

HeartQol	Physical subscale	Emotional subscale	*p*-value^#^
Total cohort (*N* = 265)			
SF-36 PCS	.68**	.14*	< .001
SF-36 MCS	.38**	.74**	< .001
*p*-value^#^	< .001	< .001	
AP (*N* = 80)			
SF-36 PCS	.78**	.23*	< .001
SF-36 MCS	.30**	.71**	< .001
*p*-value^#^	< .001	< .001	
MI (*N *= 111)			
SF-36 PCS	.62**	.14	< .001
SF-36 MCS	.44**	.76**	< .001
*p*-value^#^	.032	< .001	
HF (*N *= 74)			
SF-36 PCS	.69**	.04	< .001
SF-36 MCS	.41**	.76**	< .001
*p*-value^#^	.007	< .001	

Strong Pearson correlation coefficients *r *≥ .50 are bold

*SF-36* Short Form-36 Health Survey, *N* number of patients, *PCS* physical component summary, *MCS* mental component summary, *AP* angina, *MI* myocardial infarction, *HF* ischemic heart failure

*Correlation coefficient *p *< .05

**Correlation coefficient *p *< .001

^*#*^Steiger’s test for comparing Pearson correlation coefficients
- Discriminative validity was partially confirmed for age, sex, and disease severity, largely confirmed for the SF-36 health status and transition, and totally confirmed for anxiety, depression, and general distress (Table 5).

**Table 5:** Discriminative validity of the German HeartQoL

	HeartQoL		
	Physical subscale	Emotional subscale	Global scale
**Total cohort**			
*Age*			
Young (< 60 years; *N *= 107)	1.94 [1.80–2.07]	**2.07** [1.92–2.22]	1.98 [1.85–2.09]
Middle age (60–70 years; *N *= 99)	1.81 [1.66–1.95]	2.30 [2.15–2.46]	1.94 [1.80–2.06]
Elderly (> 70 years; *N *= 96)	1.68 [1.56–1.84]	**2.35** [2.16–2.45]^c^	1.86 [1.75–2.00]
*p*-value		*p *< .05	
*Sex*			
Female (*N *= 65)	1.79 [1.61–1.96]	**2.06** [1.84–2.22]	1.86 [1.70–2.02]
Male (*N *= 237)	1.82 [1.73–1.91]	**2.27** [2.16–2.34]	1.95 [1.86–2.02]
*p*-value		*p *< .05	
*SF-36 general health status*			
Excellent/very good (*N *= 34)	**2.23** [2.00–2.51]	**2.62** [2.44–2.75]	**2.34** [2.16–2.55]
Good (*N *= 187)	**1.97** [1.87–2.05]^b^	**2.39** [2.26–2.45]^b^	**2.08** [1.99–2.15]^b^
Fair/poor (*N *= 82)	**1.31** [1.16–1.45]^c^	**1.71** [1.52–1.87]^c^	**1.43** [1.29–1.54]^c^
*p*-value	*p *< .001	*p *< .001	*p *< .001
*SF-36 health transition*			
Improve (*N *= 69)	**1.90** [1.75–2.10]	**2.41** [2.22–2.60]	**2.03** [1.91–2.19]
No change (*N *= 80)	**2.17** [2.03–2.29]^b^	2.34 [2.19–2.45]	**2.22** [2.09–2.32]^b^
Deteriorate (*N *= 152)	**1.59** [1.48–1.70]^c^	**2.10** [1.94–2.21]^c^	**1.74** [1.62–1.83]^c^
*p*-value	b: *p *< .001; c: *p *< .05	*p *< .05	b: *p *< .001; c: *p *< .05
HADS*			
Anxious (*N *= 92)	**1.53** [1.41–1.69]	**1.65** [1.50–1.78]	**1.56** [1.45–1.70]
Not anxious (*N *= 208)	**1.94** [1.83–2.02]	**2.45** [2.37–2.54]	**2.09** [2.00–2.16]
*p*-value	*p *< .001	*p *< .001	*p *< .001
Depressive (*N *= 63)	**1.36** [1.19–1.55]	**1.49** [1.32–1.65]	**1.40** [1.25–1.56]
Not depressive (*N *= 237)	**1.93** [1.85–2.03]	**2.41** [2.32–2.47]	**2.07** [2.00–2.14]
*p*-value	*p *< .001	*p *< .001	*p *< .001
Generally stressed (*N *= 116)	**1.53** [1.88–2.09]	**1.70** [1.59–1.82]	**1.58** [1.47–1.70]
Not generally stressed (*N *= 184)	**1.99** [1.40–1.67]	**2.55** [2.46–2.64]	**2.14** [2.06–2.23]
*p*-value	*p *< .001	*p *< .001	*p *< .001
**Angina**			
*Age*			
Young (< 60 years; *N *= 23)	**1.86** [1.58–2.14]	2.12 [1.83–2.41]	1.93 [1.68–2.19]
Middle age (60–70 years; *N *= 42)	1.77 [1.52–2.01]	2.13 [1.91–2.35]	1.87 [1.66–2.08]
Elderly (> 70 years; *N *= 34)	**1.46** [1.25–1.68]^c^	2.27 [1.98–2.55]	1.70 [1.50–1.89]
*p*-value	*p *< .05		
*Sex*			
Female (*N *= 24)	1.60 [1.30–1.96]	2.14 [1.71–2.45]	1.72 [1.42–2.01]
Male (*N *= 76)	1.68 [1.50–1.83]	2.23 [2.09–2.42]	1.83 [1.70–1.99]
*SF-36 general health status*			
Excellent/very good (*N *= 16)	**2.15** [1.88–2.48]	**2.61** [2.13–2.86]	**2.28** [2.06–2.51]
Good (*N *= 61)	1.71 [1.55–1.91]	**2.35** [2.19–2.55]^b^	**1.89** [1.74–2.04]^b^
Fair/poor (*N *= 23)	**1.22** [.93–1.44]^c^	**1.60** [1.27–1.90]^c^	**1.33** [1.10–1.50]^c^
*p*-value	*p *< .05	*p *< .05	*p *< .05
*SF-36 health transition*			
Improve (*N *= 21)	**1.40** [1.17–1.78]^a^	2.40 [2.05–2.73]	1.65 [1.41–1.98]
No change (*N *= 33)	**2.05** [1.79–2.23]^b^	2.25 [2.00–2.41]	**2.11** [1.88–2.26]^b^
Deteriorate (*N *= 46)	**1.49** [1.30–1.68]	2.10 [1.85–2.33]	**1.66** [1.48–1.85]
*p*-value	*p *< .05		*p *< .05
HADS*			
Anxious (*N *= 35)	**1.48** [1.27–1.73]	**1.69** [1.47–1.85]	**1.54** [1.35–1.74]
Not anxious (*N *= 64)	**1.73** [1.57–1.94]	**2.46** [2.28–2.62]	**1.93** [1.80–2.11]
*p*-value	*p *< .05	*p *< .001	*p *< .001
Depressive (*N *= 26)	**1.22** [.95–1.41]	**1.61** [1.39–1.93]	**1.33** [1.08–1.56]
Not depressive (*N *= 73)	**1.79** [1.59–1.91]	**2.41** [2.24–2.54]	**1.96** [1.78–2.12]
*p*-value	*p *< .05	*p *< .001	*p *< .001
Generally stressed (*N *= 47)	**1.47** [1.26–1.68]	**1.78** [1.60–1.96]	**1.55** [1.37–1.73]
Not generally stressed (*N *= 52)	**1.80** [1.61–1.99]	**2.57** [2.40–2.73]	**2.01** [1.84–2.17]
*p*-value	*p *< .05	*p *< .001	*p *< .001
*Disease severity*			
CCS grade II (*N *= 57)	**1.89** [1.72–2.10]	**2.41** [2.24–2.54]	**2.03** [1.89–2.20]
CCS grade III/IV (*N *= 42)	**1.33** [1.18–1.55]	**1.92** [1.60–2.13]	**1.50** [1.34–1.67]
*p*-value	*p *< .001	*p *< .05	*p *< .001
**Myocardial infarction**			
*Age*			
Young (< 60 years; *N *= 52)	2.09 [1.86–2.26]	**2.11** [1.88–2.35]^a^	2.10 [1.89–2.27]
Middle age (60–70 years; *N *= 31)	1.94 [1.72–2.27]	**2.53** [2.27–2.84]	2.11 [1.80–2.26]
Elderly (> 70 years; *N *= 39)	1.84 [1.61–2.08]	2.35 [2.10–2.61]	1.99 [1.74–2.15]
*p*-value		*p *< .05	
*Sex*			
Female (*N *= 31)	1.95 [1.68–2.23]	2.16 [1.82–2.40]	2.01 [1.75–2.25]
Male (*N *= 92)	1.98 [1.83–2.13]	2.33 [2.18–2.50]	2.08 [1.95–2.22]
*SF-36 general health status*			
Excellent/very good (*N *= 12)	**2.10** [1.56–2.72]	**2.64** [2.40–2.88]	**2.24** [1.83–2.73]
Good (*N *= 80)	**2.14** [1.96–2.22]^b^	**2.39** [2.24–2.53]^b^	**2.22** [2.06–2.29]^b^
Fair/poor (*N *= 31)	**1.49** [1.20–1.74]^c^	**1.73** [1.41–2.04]^c^	**1.58** [1.29–1.79]^c^
*p*-value	b: *p *< .001; c: *p *< .05	*p *< .001	b: *p *< .001; c: *p *< .05
*SF-36 health transition*			
Improve (*N *= 22)	**2.33** [2.00–2.64]	2.51 [2.21–2.74]	**2.38** [2.06–2.51]
No change (*N *= 29)	**2.39** [2.10–2.60]^b^	2.44 [2.20–2.67]	**2.41** [2.21–2.56]^b^
Deteriorate (*N *= 71)	**1.71** [1.57–1.90]^c^	2.17 [1.99–2.37]	**1.85** [1.65–1.96]^c^
*p*-value	b: *p *< .001; c: *p *< .05		*p *< .05
HADS^*^			
Anxious (*N *= 33)	**1.69** [1.46–1.95]	**1.64** [1.43–1.91]	**1.68** [1.46–1.93]
Not anxious (*N *= 89)	**2.08** [1.92–2.22]	**2.52** [2.37–2.65]	**2.21** [2.02–2.28]
*p*-value	*p *< .05	*p *< .001	*p *< .001
Depressive (*N *= 22)	**1.61** [1.26–2.20]	**1.58** [1.26–1.89]	**1.61** [1.30–1.86]
Not depressive (*N *= 100)	**2.05** [1.91–2.17]	**2.43** [2.29–2.52]	**2.16** [2.03–2.25]
*p*-value	*p *< .05	*p *< .001	*p *< .001
Generally stressed (*N *= 42)	**1.66** [1.44–1.89]	**1.76** [1.54–1.97]	**1.69** [1.50–1.89]
Not generally stressed (*N *= 80)	**2.15** [1.99–2.31]	**2.57** [2.42–2.72]	**2.27** [2.13–2.41]
*p*-value	*p *< .001	*p *< .001	*p *< .001
**Ischemic heart failure**			
*Age*			
Young (< 60 years; *N *= 32)	1.75 [1.52–2.01]	1.92 [1.71–2.22]	1.80 [1.61–2.04]
Middle age (60–70 years; *N *= 26)	1.77 [1.48–2.06]	2.30 [2.00–2.61]	1.92 [1.66–2.18]
Elderly (> 70 years; *N *= 23)	1.84 [1.56–2.21]	2.35 [2.06–2.67]	1.99 [1.73–2.31]
*Sex*			
Female (*N *= 10)	1.77 [1.40–2.13]	1.63 [1.00–2.25]	1.73 [1.37–2.09]
Male (*N *= 69)	1.79 [1.62–1.98]	2.25 [2.11–2.44]	1.92 [1.78–2.09]
*SF-36 general health status*			
Excellent/very good (*N *= 6)	**2.67** [2.06–3.28]^a^	**2.67** [2.21–3.13]	**2.67** [2.23–3.10]^a^
Good (*N *= 46)	**2.01** [1.89–2.17]^b^	**2.37** [2.20–2.57]^b^	**2.11** [2.01–2.26]^b^
Fair/poor (*N *= 28)	**1.19** [.98–1.45]^c^	**1.69** [1.44–2.04]^c^	**1.33** [1.15–1.58]^c^
*p*-value	a: *p *< .05; b,c: *p *< .001	*p *< .05	a: *p *< .05; b,c: *p *< .001
*SF-36 health transition*			
Improve (*N *= 26)	**1.97** [1.72–2.23]	2.31 [2.11–2.56]	**2.07** [1.87–2.28]
No change (*N *= 18)	**2.10** [1.81–2.40]^b^	2.35 [2.04–2.65]	**2.17** [1.90–2.44]^b^
Deteriorate (*N *= 35)	**1.48** [1.27–1.75]^c^	1.96 [1.68–2.18]	**1.62** [1.43–1.87]^c^
*p*-value	*p *< .05		*p *< .05
HADS*			
Anxious (*N *= 24)	**1.42** [1.14–1.70]	**1.58** [1.29–1.87]	**1.47** [1.22–1.71]
Not anxious (*N *= 55)	**1.96** [1.78–2.14]	**2.44** [2.29–2.61]	**2.10** [1.95–2.24]
*p*-value	*p *< .05	*p *< .001	*p *< .001
Depressive (*N *= 15)	**1.29** [.93–1.66]	**1.16** [.86–1.46]	**1.26** [.96–1.55]
Not depressive (*N *= 64)	**1.90** [1.73–2.07]	**2.40** [2.26–2.55]	**2.04** [1.91–2.18]
*p*-value	*p *< .05	*p *< .001	*p *< .001
Generally stressed (*N *= 27)	**1.47** [1.19–1.74]	**1.54** [1.31–1.78]	**1.49** [1.26–1.72]
Not generally stressed (*N *= 52)	**1.95** [1.75–2.14]	**2.48** [2.32–2.65]	**2.10** [1.94–2.26]
*p*-value	*p *< .05	*p *< .001	*p *< .001
*Disease severity*			
NYHA grade II (*N *= 38)	1.84 [1.65–2.04]	2.28 [2.04–2.53]	1.97 [1.79–2.15]
NYHA grade III/IV (*N *= 40)	1.73 [1.49–1.00]	2.06 [1.84–2.31]	1.82 [1.62–2.06]

Mean comparisons with ANCOVAs (scores adjusted for age, sex, risk factors, and disease severity within diagnosis) post hoc Bonferroni correction; significant differences are bold

Age: ^a^young vs. middle age, ^b^middle age vs. elderly, ^c^young vs. elderly

SF-36 general health status: ^a^excellent/very good vs. good, ^b^good vs. fair/poor, ^c^excellent/very good vs. fair/poor

SF-36 health transition: ^a^improve vs. no change, ^b^no change vs. deteriorate, ^c^improve vs. deteriorate

*N* number of patients; [95% confidence interval], *SF-36* Short Form-36 Health Survey, *HADS* Hospital Anxiety and Depression Scale, *CCS* Canadian Cardiovascular Society, *NYHA* New York Heart Association

*HADS cut-off score ≥ 8 for the anxiety/depression subscales and ≥ 12 for the general distress factor

  *Age:* HRQL score differences were significant on the physical subscale in patients with angina with better HRQL in young patients and on the emotional subscale in the total cohort with higher HRQL in elderly patients and in patients with MI with better HRQL in middle-aged patients.
  *Sex*: HRQL score differences were only significant in the total cohort on the emotional subscale with higher HRQL in males.
  *SF*-*36 health status:* Global HRQL score differences were always significant with higher HRQL in the total cohort and each diagnosis when patients reported excellent/very good health or good health on the SF-36 health status item when compared to patients reporting fair/poor health. Other HRQL score differences on the physical and emotional subscales are detailed in Table 5.
  *SF*-*36 health transition*: The HRQL score differences were not as consistent with the SF-36 health transition item as with the health status item. However, patients in the total cohort and each diagnosis reporting either improved health or no change in health always had higher physical and global HRQL than patients reporting deteriorated health. Other HRQL score differences on the physical and emotional subscales are detailed in Table 5.
  *Anxiety, depression, and general distress scores:* HRQL score differences were significant on each scale in the total cohort as well as in each diagnosis with higher HRQL in patients who did not report anxiety or depression scores exceeding the cut-off ≥ 8 or general distress scores ≥ 12.
  *Disease severity*: HRQL score differences were significant on each scale in patients with angina with better HRQL in patients assigned to CCS grade II whereas no HRQL score differences were found in patients with ischemic heart failure.

#### Responsiveness

The HeartQoL physical subscale and the global scale means and the SF-36 PCS means improved significantly after both PCI and CR (Table 6). Significant improvement on the HeartQoL emotional subscale and the SF-36 MCS was only achieved with CR (*p *< .001). Effect sizes ranged from .31 (HeartQoL physical and global score with PCI) to .72 (HeartQoL global score with CR). The three HeartQoL effect sizes were greater with CR than those with PCI.

**Table 6:** Responsiveness of the German HeartQoL and the SF-36 component measures

HeartQol	PCI (*N *= 97)	CR (*N *= 87)
*Physical subscale*		
Baseline	1.75 ± .78	1.86 ± .71
Follow-up	1.96 ± .72	2.27 ± .58
*p*-value for change	.002	< .001
Effect size (SRM)	.31	.68
*Emotional subscale*		
Baseline	2.24 ± .73	2.25 ± .71
Follow-up	2.35 ± .73	2.55 ± .60
*p*-value for change	n.s.	< .001
Effect size (SRM)		.48
*Global scale*		
Baseline	1.89 ± .70	1.96 ± .63
Follow-up	2.07 ± .66	2.35 ± .52
*p*-value for change	.002	< .001
Effect size (SRM)	.31	.72
**SF-36 PCS**		
Baseline	39.49 ± 10.38	41.88 ± 10.31
Follow-up	43.85 ± 10.08	46.11 ± 8.73
*p*-value for change	< .001	< .001
Effect size (SRM)	.61	.51
**SF-36 MCS**		
Baseline	49.19 ± 10.78	45.96 ± 11.45
Follow-up	50.80 ± 10.77	51.03 ± 9.95
*p*-value for change	n.s.	< .001
Effect size (SRM)		.55

*PCI* percutaneous coronary intervention, *N* number of patients, *CR* cardiac rehabilitation, *scale* mean ± standard deviation, *SRM* standardized response mean, *n.s.* not significant, *SF-36* Short Form-36 Health Survey, *PCS* physical component summary, *MCS* mental component summary

## Discussion

The German HeartQoL is a valid, reliable, and responsive HRQL instrument and these data support its potential use for clinical practice and research to assess and compare HRQL in German-speaking IHD patients. Moreover, the shortness of the HeartQoL may prove to be helpful in clinical practice. The psychometric properties were evaluated based on a sample of 305 patients with either angina, MI, or ischemic heart failure from Austria and Switzerland and are consistent with the original validation study [19], the English HeartQoL version based on patients in the USA [27], the EuroAspire IV study [28], and also with validation studies in patients with atrial fibrillation [29], with an implantable cardioverter defibrillator [30] or following valve surgery [31]. According to Mokken analysis, the German HeartQoL factor structure is consistent with the original two-factor structure [18] although the moderate *H*_*i*_ coefficient loading of .48 for the eighth item in the German HeartQoL (“feeling tired, fatigued, low on energy”) reflects ambiguous wording and may need substantiation in a future study as loadings in the original study [19] and the more recent English [27], Chinese [submitted], mixed European countries [28], and Danish [29, 30] studies were all > .50. However, despite the weakness of the eighth item, the current Mokken analysis results suggest that subscales of physical and emotional HRQL are more substantial than the overall global scale, as some items had clearly weak *H*_*i*_ coefficients (< .50) on the global scale.

The German HeartQoL demonstrated adequate convergent and divergent validity as well as internal consistency reliability in the total cohort and in each diagnosis. These results are similar to other studies using the HeartQoL, e.g., the English validation study including patients with angina or MI [27] which reported strong correlations with the respective matching physical and emotional scales of the HeartQoL and SF-36 (all coefficients *r *> .60). In the original HeartQoL study [19], Cronbach’s alpha for the physical, emotional, and global scale was .90, .81, and .91, respectively, which was confirmed with the German HeartQoL version in this study. Discriminative validity was largely confirmed for the SF-36 health status and transition as well as the HADS anxiety/depression scales and the general distress factor although with age and sex the hypothesized lower scores in females as well as in older patients were not consistently met. Although there were no HeartQoL floor effects, high ceiling effects were observed on the emotional subscale in all groups with minimal effects on the physical and global scales. These observations, consistent with the original [19] and other validation studies [27–31], suggest that it may be more difficult to assess improvement in emotional HRQL than either physical or global HRQL with the HeartQoL questionnaire. Responsiveness was confirmed with significant pre-post HeartQoL physical, emotional, and global scale score changes with CR and with significant physical and global scale score changes after PCI. These results support the assumption that an invasive functional intervention such as PCI is more likely to reduce physical limitations than emotional burden. On the other hand, comprehensive rehabilitation interventions such as CR are more likely to positively influence IHD patients on a number of different levels, e.g., physical activity, heart-healthy nutrition, psychological care, relaxation, and social support, leading to a broader effect.

Regarding the discriminant validity of the German HeartQoL and contrary to our expectation, angina patients had significantly lower physical HRQL levels than patients with ischemic heart failure. This might be explained on the basis of the sample composition or that the patients with angina were significantly older than patients with MI or ischemic heart failure. Therefore, the lower HRQL scores seem to be less influenced by diagnosis but rather by age. Another explanation of this unexpected finding could be the timing of the assessment as patients with angina could have been recruited during an acute phase whereas patients with ischemic heart failure were chronic and already on an optimized medical treatment schema.

### Limitations and future research

A general critique for any “short-form” questionnaire with a relatively small number of items per scale is that the breadth of an assessment of physical or emotional HRQL may be limited. The eighth item in the HeartQoL seems to be linguistically ambiguous in the Chinese, Danish, English, and German versions as data demonstrate it may belong to either the physical or the emotional subscale. It would be worthwhile addressing this question empirically by comparing the HeartQoL head to head with other core heart disease HRQL instruments such as the MacNew. Further limitations of the German HeartQoL refer to the missing test–retest reliability and the quite small sample size of some sub-group analyses (e.g., only 10 female patients with ischemic heart failure). However, the psychometric analyses reveal that the German HeartQoL has adequate reliability and validity and is a responsive IHD-specific core HRQL instrument demonstrating its potential in research projects where economy of instruments is at a premium. Future studies will need to confirm these results using a confirmatory approach in a different sample.

### Conclusion

Psychometric characteristics of the 14-item, two-factor German version of the IHD-specific core HeartQoL questionnaire with a physical and an emotional subscale in German-speaking patients with IHD and its three major diagnoses (angina, MI, and ischemic heart failure) were examined. The German HeartQoL demonstrated excellent internal consistency reliability, adequate convergent, divergent, and discriminative validity as well as good responsiveness. Overall, the German HeartQoL can be strongly recommended for clinicians and researchers to assess and compare the impact of IHD on patients’ HRQL in German-speaking countries. The shortness of the tool may prove to be helpful in clinical practice too.

## Data Availability

The datasets used and/or analyzed during the current study are available from the corresponding author on reasonable request.

## References

[1] Anker SD, Agewall S, Borggrefe M, Calvert M, Caro JJ, Cowie MR (2014). The importance of patient-reported outcomes: A call for their comprehensive integration in cardiovascular clinical trials. European Heart Journal.

[2] Rumsfeld JS, Alexander KP, Goff DC, Graham MM, Ho PM, Masoudi FA (2013). Cardiovascular health: The importance of measuring patient-reported health status. Circulation.

[3] McGee HM, Oldridge N, Hellemans IM (2005). Quality of life evaluation in cardiovascular disease: A role for the European Society of Cardiology? (Editorial). European Journal of Cardiovascular Prevention and Rehabilitation.

[4] World Health Organization. (2018). *Global health estimates 2016: Deaths by cause, age, sex, by country and BY Region, 2000*–*2016*. Geneva: World Health Organization. Retrieved September 11, 2018, from http://www.who.int/healthinfo/global_burden_disease/estimates/en/.

[5] Statistik Austria. G*estorbene 2017 nach Todesursachen, Alter und Geschlecht [Deceased 2017 by cause of death, age and sex].* Retrieved September 11, 2018, from https://www.statistik.at/web_de/statistiken/menschen_und_gesellschaft/gesundheit/todesursachen/todesursachen_im_ueberblick/index.html.

[6] Statistisches Bundesamt DeStatis Deutschland. *Todesursachen in Deutschland [Causes of death in Germany in 2015].* Fachserie 12, Reihe 4. Retrieved September 11, 2018, from https://www.destatis.de/DE/Publikationen/Thematisch/Gesundheit/Todesursachen/Todesursachen.html.

[7] Bundesamt für Statistik, Schweizerische Eidgenossenschaft. *Spezifische Todesursachen in 2015 [Specific causes of death in 2015]*. Retrieved September 11, 2018, from https://www.bfs.admin.ch/bfs/de/home/statistiken/gesundheit/gesundheitszustand/sterblichkeit-todesursachen/spezifische.html.

[8] Murray CJL, Vos T, Lozano R, Naghavi M, Flaxman AD, Michaud C (2012). Disability-adjusted life years (DALYs) for 291 diseases and injuries in 21 regions, 1990–2010: A systematic analysis for the Global Burden of Disease Study 2010. The Lancet.

[9] Griebler, R., Anzenberger, J., & Eisenmann, A. (2014). *Herz*-*Kreislauf*-*Erkrankungen in Österreich: Angina Pectoris, Myokardinfarkt, ischämischer Schlaganfall, periphere arterielle Verschlusskrank*-*heit. Epidemiologie und Prävention. [Cardiovascular diseases in Austria: Angina pectoris, myocardial infarction, ischaemic stroke, arterial obstructive disease. Epidemiology and prevention].* Wien: Bundesministerium für Gesundheit.

[10] Höfer S, Benzer W, Oldridge N (2014). Change in health-related quality of life in patients with coronary artery disease predicts 4-year mortality. International Journal of Cardiology.

[11] Benzer W, Phillippi A, Höfer S, Friedrich O, Oldridge N (2016). Health-related quality of life predicts unplanned rehospitalization following coronary revascularisation. Herz.

[12] Ware JE, Kosinski M (2005). SF-36^®^ Physical and Mental Health Summary Scales: A manual for users of version 1.

[13] Ware JE, Gandek B (1998). Overview of the SF-36 Health Survey and the International Quality of Life Assessment (IQOLA) Project. Journal of Clinical Epidemiology.

[14] Alonso J, Ferrer M, Gandek B, Ware J, Aaronson NK, Mosconi P (2004). Health-related quality of life associated with chronic conditions in eight countries: Results from the International Quality of Life Assessment (IQOLA) Project. Quality of Life Research.

[15] Soto M, Failde I, Márquez S, Benítez E, Ramos I, Barba A (2005). Physical and mental component summaries score of the SF-36 in coronary patients. Quality of Life Research.

[16] Mommersteeg PM, Denollet J, Spertus JA, Pedersen SS (2009). Health status as a risk factor in cardiovascular disease: A systematic review of current evidence. American Heart Journal.

[17] Testa MA, Simonson DC (1996). Assessment of quality of life outcomes. The New England Journal of Medicine.

[18] Oldridge N, Höfer S, McGee H, Conroy R, Doyle F, Saner H (2014). The HeartQoL: Part I. Development of a new core health-related quality of life questionnaire for patients with ischemic heart disease. European Journal of Preventive Cardiology.

[19] Oldridge N, Höfer S, McGee H, Conroy R, Doyle F, Saner H (2014). The HeartQoL: Part II. Validation of a new core health-related quality of life questionnaire for patients with ischemic heart disease. European Journal of Preventive Cardiology.

[20] Spertus JA, Winder JA, Dewhurst TA, Deyo RA, Prodzinski J, McDonnell M (1995). Development and evaluation of the Seattle Angina Questionnaire: A new functional status measure for coronary artery disease. Journal of the American College of Cardiology.

[21] Höfer S, Lim LL, Guyatt GH, Oldridge N (2004). The MacNew Heart Disease Health-related Quality of Life Instrument: A summary. Health and Quality of Life Outcomes.

[22] Rector TS, Kubo SH, Cohn JN (1987). Patients’ self-assessment of their congestive heart failure: Part 2. Content, reliability, and validity of a new measure, the Minnesota Living with Heart Failure questionnaire. Heart Failure.

[23] Scientific Advisory Committee of the Medical Outcomes Trust (2002). Assessing health status and quality-of-life instruments: Attributes and review criteria. Quality of Life Research.

[24] Bullinger, M., & Kirchberger, I. (1998). *SF*-*36. Fragebogen zum Gesundheitszustand. [SF*-*36. Questionnaire concerning state of health].* Manual. Göttingen: Hogrefe.

[25] Höfer S, Benzer W, Schüßler G, von Steinbüchel N, Oldridge NB (2003). Health-related quality of life in patients with coronary artery disease treated for angina: Validity and reliability of German translations of two specific questionnaires. Quality of Life Research.

[26] Quittan M, Wiesinger GF, Crevenna R, Nuhr MJ, Posch M, Hülsmann M (2001). Cross-cultural adaptation of the Minnesota Living with Heart Failure Questionnaire for German-speaking patients. Journal of Rehabilitation Medicine.

[27] Oldridge N, Cho C, Randal T, Low M, Höfer S (2018). Validation of the English Version of the HeartQoL Health-Related Quality of Life Questionnaire in Patients With Coronary Heart Disease. Journal of Cardiopulmonary Rehabilitation and Prevention.

[28] De Smedt D, Clays E, Höfer S, Oldridge N, Kotseva K, Maggioni AP (2016). Validity and reliability of the HeartQoL questionnaire in a large sample of stable coronary patients: The EUROASPIRE IV Study of the European Society of Cardiology. European Journal of Preventive Cardiology.

[29] Kristensen MS, Zwisler AD, Berg SK, Zangger G, Gronset CN, Risom SS (2016). Validating the HeartQoL questionnaire in patients with atrial fibrillation. European Journal of Preventive Cardiology.

[30] Zangger G, Zwisler AD, Kikkenborg Berg S, Kristensen MS, Gronset CN, Uddin J (2018). Psychometric properties of HeartQoL, a core heart disease-specific health-related quality of life questionnaire, in Danish implantable cardioverter defibrillator recipients. European Journal of Preventive Cardiology.

[31] Grønset CN, Thygesen LC, Kikkenborg Berg S, Zangger G, Kristensen MS, Sibilitz K (2019). Measuring HRQoL following heart valve surgery: The HeartQoL questionnaire is a valid and reliable core heart disease instrument. Quality of Life Research.

[32] Herrmann-Lingen, C., Buss, U., & Snaith, R. P. (1995). *Hospital Anxiety and Depression Scale*—*deutsche Version (HADS*-*D). [Hospital Anxiety and Depression Scale*—*German version (HADS*-*D)].* Bern: Hans Huber Verlag.

[33] Campeau L (1976). Grading of angina pectoris. Circulation.

[34] Criteria Committee of the New York Heart Association (1994). Nomenclature and criteria for diagnosis of diseases of the heart and great vessels.

[35] Bjelland I, Dahl AA, Haug TT, Neckelmann D (2002). The validity of the Hospital Anxiety and Depression Scale: An updated literature review. Psychosomatic Research.

[36] Palacios JE, Khondoker M, Achilla E, Tylee A, Hotopf M (2016). A single, one-off measure of depression and anxiety predicts future symptoms, higher healthcare costs, and lower quality of life in coronary heart disease patients: Analysis from a multi-wave, Primary care cohort study. PLoS ONE.

[37] Corp IBM (2015). IBM SPSS statistics for windows, version 24.0.

[38] StataCorp (2015). Stata statistical software: Release 14.

[39] Terwee CB, Bot SDM, de Boer MR, van der Windt DAWM, Knol DL, Dekker J (2007). Quality criteria were proposed for measurement properties of health status questionnaires. Journal of Clinical Epidemiology.

[40] Meijer RR, Baneke JJ (2004). Analyzing psychopathology items: A case for nonparametric item response theory modeling. Psychological Methods.

[41] Cohen J (1988). Statistical power analysis for the behavioral sciences.

[42] Steiger JH (1980). Testing pattern hypotheses on correlation matrices: Alternative statistics and some empirical results. Multivariate Behavioural Research.

[43] Hays RD, Anderson RT, Revicki D, Staquet M, Hays RD, Fayers PM (1998). Assessing reliability and validity of measurement in clinical trials. Quality of life assessment in clinical trials.

[44] Liang MH, Fossel AH, Larson MG (1990). Comparisons of five health status instruments for orthopedic evaluation. Medical Care Journal.

[45] Griebler, R., Anzenberger, J., & Eisenmann, A. (2014). *Herz*-*Kreislauf*-*Erkrankungen in Österreich*—*Angina pectoris, Myokardinfarkt, ischämischer Schlaganfall, periphere arterielle Verschluss*-*krankheit. Epidemiologie und Prävention [Cardiovascular diseases in Austria*—*angina pectoris, myocardial infarction, ischemic stroke, peripheral artery disease. Epidemiology and prevention.]* Vienna: Federal Ministry of Health. Retrieved November 12, 2019, from https://jasmin.goeg.at/113/1/Herz-Kreislauf-Erkrankungen%20in%20%C3%96sterreich.pdf.

[46] Robert Koch-Institute. (2012). *Gesundheitsberichterstattung der Bundesrepublik Deutschland [Health Report of the Federal Republic of Germany].* Retrieved November 13, 2019, fromhttps://www.rki.de/DE/Content/Gesundheitsmonitoring/Themen/Chronische_Erkrankungen/HKK/HKK_node.html.

[47] Schweizerische Eidgenossenschaft—Bundesamt für Statistik. (2016). *Herz*-*Kreislauferkrankungen in der Schweiz [Cardiovascular diseases in Switzerland].* Retrieved November 14, 2019, fromhttps://www.bfs.admin.ch/bfs/de/home/statistiken/gesundheit/gesundheitszustand/krankheiten/herz-kreislauf-erkrankungen.html.

